# 2-[(4-Ethyl­phen­yl)imino­meth­yl]-3,5-dimethoxy­phenol

**DOI:** 10.1107/S1600536809004784

**Published:** 2009-02-18

**Authors:** Zarife Sibel Şahin, Ferda Erşahin, Ayşen Alaman Ağar, Şamil Işık

**Affiliations:** a Department of Physics, Faculty of Arts and Sciences, Ondokuz Mayıs University, Kurupelit, TR-55139 Samsun, Turkey; b Department of Chemistry, Faculty of Arts and Sciences, Ondokuz Mayıs University, 55139 Samsun, Turkey

## Abstract

The title compound, C_17_H_19_NO_3_, adopts the phenol–imine tautomeric form, with a resonance-assisted O—H⋯N intra­molecular hydrogen bond [O⋯N = 2.551 (3) Å]. The dihedral angle between the two benzene rings is 45.42 (7)°. The two meth­oxy groups are coplanar with the attached benzene ring [C—O—C—C torsion angles = −1.1 (5) and 3.2 (4)°].

## Related literature

For the photochromic and thermochromic characteristics of Schiff base compounds, see: Hadjoudis *et al.* (1987 ▶); Lozier *et al.* (1975 ▶). For the notation of hydrogen-bonding motifs, see: Bernstein *et al.* (1995 ▶).
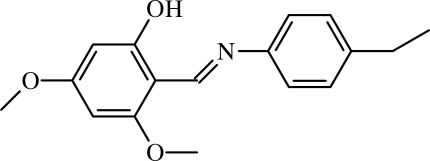


## Experimental

### 

#### Crystal data

C_17_H_19_NO_3_
*M* *_r_* = 285.33
Orthorhombic, 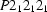

*a* = 7.5026 (5) Å
*b* = 9.4540 (8) Å
*c* = 21.4408 (13) Å
*V* = 1520.79 (19) Å^3^
*Z* = 4
Mo *K*α radiation
μ = 0.09 mm^−1^
*T* = 296 K
0.46 × 0.35 × 0.11 mm

#### Data collection

Stoe IPDS II diffractometer
Absorption correction: integration (*X-RED32*; Stoe & Cie, 2002 ▶) *T*_min_ = 0.991, *T*_max_ = 0.998
10094 measured reflections
1831 independent reflections
1036 reflections with *I* > 2σ(*I*)
*R*_int_ = 0.049

#### Refinement

*R*[*F* ^2^ > 2σ(*F* ^2^)] = 0.040
*wR*(*F* ^2^) = 0.077
*S* = 0.92
1831 reflections
191 parameters
H-atom parameters constrained
Δρ_max_ = 0.09 e Å^−3^
Δρ_min_ = −0.11 e Å^−3^

### 

Data collection: *X-AREA* (Stoe & Cie, 2002 ▶); cell refinement: *X-AREA*; data reduction: *X-RED32* (Stoe & Cie, 2002 ▶); program(s) used to solve structure: *SHELXS97* (Sheldrick, 2008 ▶); program(s) used to refine structure: *SHELXL97* (Sheldrick, 2008 ▶); molecular graphics: *ORTEP-3 for Windows* (Farrugia, 1997 ▶); software used to prepare material for publication: *WinGX* (Farrugia, 1999 ▶).

## Supplementary Material

Crystal structure: contains datablocks I, global. DOI: 10.1107/S1600536809004784/ci2762sup1.cif

Structure factors: contains datablocks I. DOI: 10.1107/S1600536809004784/ci2762Isup2.hkl

Additional supplementary materials:  crystallographic information; 3D view; checkCIF report

## Figures and Tables

**Table 1:** Hydrogen-bond geometry (Å, °)

*D*—H⋯*A*	*D*—H	H⋯*A*	*D*⋯*A*	*D*—H⋯*A*
O3—H3⋯N1	0.82	1.82	2.551 (3)	149

**Fig. 1.**
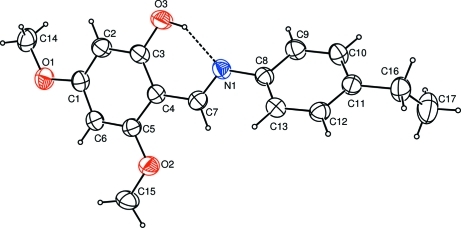
The molecular structure of the title compound, showing the atom-numbering scheme. Displacement ellipsoids are drawn at the 30% probability.

## supplementary crystallographic information

### Comment

Most Schiff base compounds have antibacterial, anticancer, anti-inflammatory and antioxic properties. In addition Schiff bases are important in diverse fields of chemistry and biochemistry owing to their biological activites (Lozier *et al.*, 1975). There are two types of intramolecular hydrogen bonds in Schiff bases which may stabilize them in keto–amine (N—H···O hydrogen bond) or phenol–imine (N···H—O hydrogen bond) tautomeric forms (Hadjoudis *et al.*, 1987). Our investigations show that the title compound adopts the phenol–imine tautomeric form (Fig. 1).

The N1—C7 bond length of 1.281 (3) Å is typical of a double bond. The dihedral angle between the C1–C6 and C8–C13 benzene rings is 45.4 (2)°. The C4—C7—N1—C8 torsion angle is -179.5 (3)°. The strong intramolecular O3—H3···N1 hydrogen bond forms an *S*(6) motif (Bernstein *et al.*, 1995).

### Experimental

2-Hydroxy-4,6-dimethoxybenzaldehyde (0.0327 g, 0.18 mmol) in ethanol (20 ml) was added to a solution of 4-ethylaniline (0.0219 g, 0.18 mmol) in ethanol (20 ml) and the reaction mixture was stirred for 1 h under reflux, to obtain the title compound. Single crystals suitable for X-ray analysis were obtained by slow evaporation of an ethanol solution (yield 61%; m.p.351–353 K).

### Refinement

All H atoms were placed in calculated positions and constrained to ride on their parent atoms, with C—H = 0.93–0.97 Å, O—H = 0.82 Å and *U*_iso_(H) = 1.2*U*_eq_(C) and 1.5*U*_eq_(C_methyl_,O). In the absence of significant anomalous dispersion effects, Friedel pairs were merged before the final refinement.

### Crystal data

**Table:**

C_17_H_19_NO_3_	*F*(000) = 608
*M**_r_* = 285.33	*D*_x_ = 1.246 Mg m^−^^3^
Orthorhombic, *P*2_1_2_1_2_1_	Mo *K*α radiation, λ = 0.71073 Å
Hall symbol: P 2ac 2ab	Cell parameters from 10094 reflections
*a* = 7.5026 (5) Å	θ = 1.9–27.7°
*b* = 9.4540 (8) Å	µ = 0.09 mm^−^^1^
*c* = 21.4408 (13) Å	*T* = 296 K
*V* = 1520.79 (19) Å^3^	Prism, yellow
*Z* = 4	0.46 × 0.35 × 0.11 mm

### Data collection

**Table:**

Stoe IPDS II diffractometer	1831 independent reflections
Radiation source: fine-focus sealed tube	1036 reflections with *I* > 2σ(*I*)
graphite	*R*_int_ = 0.049
Detector resolution: 6.67 pixels mm^-1^	θ_max_ = 26.5°, θ_min_ = 1.9°
ω scans	*h* = −9→8
Absorption correction: integration (*X-RED32*; Stoe & Cie, 2002)	*k* = −11→10
*T*_min_ = 0.991, *T*_max_ = 0.998	*l* = −26→26
10094 measured reflections	

### Refinement

**Table:**

Refinement on *F*^2^	Primary atom site location: structure-invariant direct methods
Least-squares matrix: full	Secondary atom site location: difference Fourier map
*R*[*F*^2^ > 2σ(*F*^2^)] = 0.040	Hydrogen site location: inferred from neighbouring sites
*wR*(*F*^2^) = 0.077	H-atom parameters constrained
*S* = 0.92	*w* = 1/[σ^2^(*F*_o_^2^) + (0.0332*P*)^2^] where *P* = (*F*_o_^2^ + 2*F*_c_^2^)/3
1831 reflections	(Δ/σ)_max_ = 0.001
191 parameters	Δρ_max_ = 0.09 e Å^−^^3^
0 restraints	Δρ_min_ = −0.11 e Å^−^^3^

### Special details

**Table:**

Geometry. All e.s.d.'s (except the e.s.d. in the dihedral angle between two l.s. planes) are estimated using the full covariance matrix. The cell e.s.d.'s are taken into account individually in the estimation of e.s.d.'s in distances, angles and torsion angles; correlations between e.s.d.'s in cell parameters are only used when they are defined by crystal symmetry. An approximate (isotropic) treatment of cell e.s.d.'s is used for estimating e.s.d.'s involving l.s. planes.
Refinement. Refinement of *F*^2^ against ALL reflections. The weighted *R*-factor *wR* and goodness of fit *S* are based on *F*^2^, conventional *R*-factors *R* are based on *F*, with *F* set to zero for negative *F*^2^. The threshold expression of *F*^2^ > σ(*F*^2^) is used only for calculating *R*-factors(gt) *etc*. and is not relevant to the choice of reflections for refinement. *R*-factors based on *F*^2^ are statistically about twice as large as those based on *F*, and *R*- factors based on ALL data will be even larger.

### Fractional atomic coordinates and isotropic or equivalent isotropic displacement parameters (Å^2^)

**Table:**

	*x*	*y*	*z*	*U*_iso_*/*U*_eq_	
C1	0.2906 (4)	0.3417 (3)	0.70797 (12)	0.0630 (8)	
C2	0.2290 (5)	0.3445 (3)	0.76807 (12)	0.0692 (9)	
H2	0.1988	0.4295	0.7871	0.083*	
C3	0.2128 (4)	0.2174 (3)	0.79972 (12)	0.0635 (8)	
C4	0.2526 (4)	0.0871 (3)	0.77182 (11)	0.0566 (7)	
C5	0.3141 (4)	0.0911 (3)	0.70914 (12)	0.0610 (8)	
C6	0.3354 (4)	0.2165 (3)	0.67808 (12)	0.0630 (8)	
H6	0.3794	0.2178	0.6375	0.076*	
C7	0.2409 (4)	−0.0436 (3)	0.80628 (12)	0.0601 (7)	
H7	0.2663	−0.1284	0.7862	0.072*	
C8	0.1874 (4)	−0.1759 (3)	0.89708 (11)	0.0559 (7)	
C9	0.2513 (4)	−0.1775 (3)	0.95769 (12)	0.0679 (8)	
H9	0.2991	−0.0956	0.9749	0.081*	
C10	0.2447 (5)	−0.3000 (3)	0.99269 (12)	0.0699 (8)	
H10	0.2930	−0.3004	1.0326	0.084*	
C11	0.1679 (4)	−0.4216 (3)	0.96967 (12)	0.0625 (8)	
C12	0.1013 (4)	−0.4181 (3)	0.90983 (12)	0.0634 (8)	
H12	0.0481	−0.4988	0.8934	0.076*	
C13	0.1118 (4)	−0.2971 (3)	0.87352 (11)	0.0604 (8)	
H13	0.0675	−0.2980	0.8330	0.072*	
C14	0.2705 (5)	0.5932 (3)	0.69842 (14)	0.0878 (10)	
H14A	0.2939	0.6663	0.6685	0.132*	
H14B	0.3412	0.6091	0.7350	0.132*	
H14C	0.1464	0.5944	0.7094	0.132*	
C15	0.4190 (5)	−0.0439 (4)	0.62229 (12)	0.0884 (11)	
H15A	0.4361	−0.1407	0.6102	0.133*	
H15B	0.5311	0.0049	0.6212	0.133*	
H15C	0.3372	0.0007	0.5940	0.133*	
C16	0.1567 (5)	−0.5544 (3)	1.00901 (14)	0.0857 (10)	
H16A	0.1075	−0.5299	1.0494	0.103*	
H16B	0.0747	−0.6198	0.9892	0.103*	
C17	0.3298 (6)	−0.6273 (4)	1.01865 (17)	0.1268 (16)	
H17A	0.3789	−0.6538	0.9790	0.190*	
H17B	0.3116	−0.7105	1.0435	0.190*	
H17C	0.4108	−0.5648	1.0397	0.190*	
N1	0.1964 (3)	−0.0451 (2)	0.86397 (10)	0.0629 (7)	
O1	0.3148 (3)	0.4591 (2)	0.67216 (8)	0.0834 (7)	
O2	0.3479 (3)	−0.0388 (2)	0.68417 (8)	0.0781 (6)	
O3	0.1587 (4)	0.2229 (2)	0.85970 (8)	0.0879 (7)	
H3	0.1526	0.1425	0.8738	0.132*	

### Atomic displacement parameters (Å^2^)

**Table:**

	*U*^11^	*U*^22^	*U*^33^	*U*^12^	*U*^13^	*U*^23^
C1	0.063 (2)	0.060 (2)	0.0665 (18)	−0.0127 (18)	−0.0040 (17)	0.0093 (15)
C2	0.082 (2)	0.0589 (19)	0.0672 (18)	−0.0111 (18)	0.0035 (17)	−0.0034 (15)
C3	0.070 (2)	0.0659 (18)	0.0548 (15)	−0.0106 (19)	0.0023 (15)	−0.0011 (15)
C4	0.0569 (19)	0.0566 (18)	0.0563 (15)	−0.0072 (17)	−0.0013 (14)	0.0015 (14)
C5	0.059 (2)	0.0607 (19)	0.0630 (17)	0.0015 (17)	−0.0005 (14)	−0.0030 (15)
C6	0.062 (2)	0.0680 (19)	0.0586 (15)	−0.0035 (19)	0.0001 (14)	0.0062 (16)
C7	0.0531 (18)	0.0607 (18)	0.0665 (17)	0.0020 (17)	0.0004 (15)	0.0003 (14)
C8	0.0524 (19)	0.062 (2)	0.0534 (15)	0.0002 (17)	0.0044 (14)	0.0009 (14)
C9	0.074 (2)	0.067 (2)	0.0621 (16)	−0.0129 (18)	−0.0080 (16)	−0.0051 (15)
C10	0.075 (2)	0.078 (2)	0.0569 (15)	−0.0050 (19)	−0.0071 (15)	0.0078 (16)
C11	0.061 (2)	0.062 (2)	0.0642 (17)	0.0031 (18)	0.0066 (14)	0.0035 (15)
C12	0.067 (2)	0.0563 (19)	0.0664 (18)	−0.0047 (17)	0.0065 (16)	−0.0045 (15)
C13	0.059 (2)	0.068 (2)	0.0541 (15)	−0.0018 (17)	0.0005 (14)	−0.0004 (16)
C14	0.104 (3)	0.062 (2)	0.097 (2)	−0.006 (2)	−0.009 (2)	0.0148 (18)
C15	0.110 (3)	0.092 (2)	0.0636 (18)	0.001 (2)	0.0264 (18)	−0.0078 (17)
C16	0.100 (3)	0.076 (2)	0.081 (2)	0.003 (2)	0.0076 (19)	0.0194 (18)
C17	0.125 (4)	0.119 (3)	0.137 (3)	0.047 (3)	0.038 (3)	0.058 (3)
N1	0.0689 (18)	0.0643 (15)	0.0554 (14)	−0.0059 (15)	0.0017 (12)	0.0050 (11)
O1	0.1059 (19)	0.0655 (13)	0.0788 (14)	−0.0060 (14)	0.0029 (12)	0.0146 (12)
O2	0.0968 (17)	0.0701 (14)	0.0674 (12)	0.0057 (13)	0.0197 (12)	0.0004 (11)
O3	0.136 (2)	0.0651 (13)	0.0621 (11)	−0.0094 (16)	0.0212 (12)	−0.0037 (9)

### Geometric parameters (Å, °)

**Table:**

C1—O1	1.362 (3)	C11—C12	1.377 (3)
C1—C2	1.369 (3)	C11—C16	1.515 (4)
C1—C6	1.387 (4)	C12—C13	1.386 (4)
C2—C3	1.385 (4)	C12—H12	0.93
C2—H2	0.93	C13—H13	0.93
C3—O3	1.350 (3)	C14—O1	1.426 (3)
C3—C4	1.401 (3)	C14—H14A	0.96
C4—C5	1.421 (3)	C14—H14B	0.96
C4—C7	1.442 (3)	C14—H14C	0.96
C5—O2	1.364 (3)	C15—O2	1.430 (3)
C5—C6	1.370 (4)	C15—H15A	0.96
C6—H6	0.93	C15—H15B	0.96
C7—N1	1.281 (3)	C15—H15C	0.96
C7—H7	0.93	C16—C17	1.485 (5)
C8—C13	1.375 (4)	C16—H16A	0.97
C8—C9	1.385 (3)	C16—H16B	0.97
C8—N1	1.428 (3)	C17—H17A	0.96
C9—C10	1.381 (4)	C17—H17B	0.96
C9—H9	0.93	C17—H17C	0.96
C10—C11	1.377 (4)	O3—H3	0.82
C10—H10	0.93		
			
O1—C1—C2	124.0 (3)	C11—C12—H12	119.2
O1—C1—C6	113.7 (2)	C13—C12—H12	119.2
C2—C1—C6	122.2 (3)	C8—C13—C12	120.3 (2)
C1—C2—C3	118.3 (3)	C8—C13—H13	119.8
C1—C2—H2	120.9	C12—C13—H13	119.8
C3—C2—H2	120.9	O1—C14—H14A	109.5
O3—C3—C2	117.4 (3)	O1—C14—H14B	109.5
O3—C3—C4	120.3 (3)	H14A—C14—H14B	109.5
C2—C3—C4	122.3 (2)	O1—C14—H14C	109.5
C3—C4—C5	116.7 (3)	H14A—C14—H14C	109.5
C3—C4—C7	121.4 (2)	H14B—C14—H14C	109.5
C5—C4—C7	121.8 (3)	O2—C15—H15A	109.5
O2—C5—C6	124.5 (2)	O2—C15—H15B	109.5
O2—C5—C4	114.1 (2)	H15A—C15—H15B	109.5
C6—C5—C4	121.4 (3)	O2—C15—H15C	109.5
C5—C6—C1	119.0 (3)	H15A—C15—H15C	109.5
C5—C6—H6	120.5	H15B—C15—H15C	109.5
C1—C6—H6	120.5	C17—C16—C11	114.4 (3)
N1—C7—C4	121.3 (3)	C17—C16—H16A	108.7
N1—C7—H7	119.3	C11—C16—H16A	108.7
C4—C7—H7	119.3	C17—C16—H16B	108.7
C13—C8—C9	118.6 (3)	C11—C16—H16B	108.7
C13—C8—N1	124.0 (2)	H16A—C16—H16B	107.6
C9—C8—N1	117.3 (3)	C16—C17—H17A	109.5
C10—C9—C8	120.4 (3)	C16—C17—H17B	109.5
C10—C9—H9	119.8	H17A—C17—H17B	109.5
C8—C9—H9	119.8	C16—C17—H17C	109.5
C11—C10—C9	121.4 (3)	H17A—C17—H17C	109.5
C11—C10—H10	119.3	H17B—C17—H17C	109.5
C9—C10—H10	119.3	C7—N1—C8	120.1 (2)
C10—C11—C12	117.8 (3)	C1—O1—C14	118.1 (2)
C10—C11—C16	121.0 (3)	C5—O2—C15	117.6 (2)
C12—C11—C16	121.2 (3)	C3—O3—H3	109.5
C11—C12—C13	121.5 (3)		
			
O1—C1—C2—C3	−179.1 (3)	N1—C8—C9—C10	179.2 (3)
C6—C1—C2—C3	0.5 (5)	C8—C9—C10—C11	−2.9 (5)
C1—C2—C3—O3	177.4 (3)	C9—C10—C11—C12	1.5 (5)
C1—C2—C3—C4	−1.8 (5)	C9—C10—C11—C16	−178.3 (3)
O3—C3—C4—C5	−178.1 (3)	C10—C11—C12—C13	0.4 (4)
C2—C3—C4—C5	1.1 (4)	C16—C11—C12—C13	−179.7 (3)
O3—C3—C4—C7	−1.4 (4)	C9—C8—C13—C12	−0.4 (4)
C2—C3—C4—C7	177.8 (3)	N1—C8—C13—C12	−177.1 (3)
C3—C4—C5—O2	−179.0 (3)	C11—C12—C13—C8	−1.0 (4)
C7—C4—C5—O2	4.3 (4)	C10—C11—C16—C17	−71.9 (4)
C3—C4—C5—C6	0.8 (4)	C12—C11—C16—C17	108.2 (4)
C7—C4—C5—C6	−175.8 (3)	C4—C7—N1—C8	−179.5 (3)
O2—C5—C6—C1	177.8 (3)	C13—C8—N1—C7	−43.7 (4)
C4—C5—C6—C1	−2.1 (4)	C9—C8—N1—C7	139.6 (3)
O1—C1—C6—C5	−179.0 (3)	C2—C1—O1—C14	−1.1 (5)
C2—C1—C6—C5	1.4 (5)	C6—C1—O1—C14	179.3 (3)
C3—C4—C7—N1	−1.1 (4)	C6—C5—O2—C15	3.2 (4)
C5—C4—C7—N1	175.4 (3)	C4—C5—O2—C15	−176.9 (3)
C13—C8—C9—C10	2.3 (5)		

### Hydrogen-bond geometry (Å, °)

**Table:**

*D*—H···*A*	*D*—H	H···*A*	*D*···*A*	*D*—H···*A*
O3—H3···N1	0.82	1.82	2.551 (3)	149
